# Longitudinal associations between cardiovascular biomarkers and metabolic syndrome during puberty: the PUBMEP study

**DOI:** 10.1007/s00431-022-04702-6

**Published:** 2022-11-15

**Authors:** Esther M. González-Gil, Augusto Anguita-Ruiz, Anton Kalén, Carmela De las Lamas Perez, Azahara I. Rupérez, Rocio Vázquez-Cobela, Katherine Flores, Angel Gil, Mercedes Gil-Campos, Gloria Bueno, Rosaura Leis, Concepción M. Aguilera

**Affiliations:** 1 Department of Biochemistry and Molecular Biology II, Center of Biomedical Research (CIBM), Instituto de Nutrición Y Tecnología de los Alimentos, Universidad de Granada, Granada, Spain; 2 GENUD (Growth, Exercise, NUtrition and Development) Research Group, Universidad de Zaragoza, Zaragoza, Spain; 3 CIBEROBN (Physiopathology of Obesity and Nutrition Network), Institute of Health Carlos III (ISCIII), Madrid, Spain; 4 Instituto de Investigación Biosanitaria IBS, Granada, Spain; 5 Unit of Investigation in Nutrition, Growth and Human Development of Galicia, Pediatric Department, Clinic University Hospital of Santiago, University of Santiago de Compostela, Santiago de Compostela, Spain; 6 Metabolism and Investigation Unit, Reina Sofia University Hospital, Maimónides Institute of Biomedicine Research of Córdoba (IMIBIC), University of Córdoba, Córdoba, Spain; 7 Pediatric Endocrinology Unit, Facultad de Medicina, Clinic University Hospital Lozano Blesa, Universidad de Zaragoza, Zaragoza, 50009 Spain

**Keywords:** Puberty, Metabolic syndrome, Inflammation, Cardiovascular risk

## Abstract

Puberty has been described as a life stage of considerable metabolic risk specially for those with obesity. The low-grade systemic inflammatory status associated with obesity could be one of the connections with metabolic syndrome (MetS). Thus, we aimed to assess the relationship between inflammatory and cardiovascular biomarkers and the development of MetS during puberty. Seventy-five children from the PUBMEP study (33 females), aged 4–18 years, were included. Cardiovascular and inflammatory biomarkers were measured in the prepubertal and pubertal stage, including high-sensitivity C-reactive protein (CRP), leptin, tumor necrosis factor-alpha (TNFα), interleukin 8 (IL8), monocyte chemoattractant protein 1 (MCP-1), total plasminogen activator inhibitor-1 (tPAI), resistin, adiponectin, myeloperoxidase (MPO), and soluble intercellular adhesion molecule-1 (sICAM-1). MetS was diagnosed at each measurement point. Mixed-effects and logistic regressions were performed. Those children with MetS in puberty presented higher prepubertal values of several cardiometabolic biomarkers in comparison to those without MetS (z-score body mass index (zBMI), waist circumference, insulin, HOMA-IR, leptin, and tPAI (*p* < 0.05)). For prepubertal children with obesity, the odds of developing MetS in puberty were significantly higher in those having high zBMI (OR = 4.27; CI: 1.39–22.59) or high concentrations of tPAI (OR = 1.19; CI: 1.06–1.43).

*Conclusion*: Those with obesity with higher prepubertal tPAI plasma levels had 19% higher odds of having MetS at puberty highlighting the existence of association between MetS, obesity, and inflammation already in puberty. Thus, assessing cardiometabolic and inflammatory status in children with obesity already at prepuberty is key to avoiding future comorbidities.

**Table:** 

**What is Known:** *• Inflammation, metabolic syndrome, and obesity may have their onset in childhood.* *• Puberty is a life stage characterized for an increased cardiovascular risk.*	
**What is New:** *• Prepuberty state could be an early indicator of future cardiometabolic risk.* *• Children with obesity and high total plasminogen have higher odds of future metabolic syndrome.*

**Supplementary Information:**

The online version contains supplementary material available at 10.1007/s00431-022-04702-6.

## Introduction

Childhood obesity is considered a worldwide concern as the rate of increase, in many countries, has been greater than the rate of increase in adult obesity [1]. In addition, childhood obesity has been related to cardiovascular diseases and diabetes in adulthood [2]. Among these diseases, metabolic syndrome (MetS) is a cluster of several cardiometabolic risk factors such as hyperglycemia, dyslipidemia, elevated blood pressure, and central or total adiposity [3]. In 2020, about 3% of children and 5% of adolescents had MetS, with some variation across countries and regions and the definitions used [4].

The presence of MetS in childhood has been associated with the risk of cardiometabolic disorders in adulthood [5, 6] and with subclinical atherosclerosis in obese children [7]. Additionally, those with MetS in childhood showed a higher risk in later life of diabetes mellitus type 2 (T2DM) and a higher risk of having high carotid intima-media thickness cIMT [5]. Thus, the presence of MetS in childhood could predict future cardiovascular risk factors; this could be due to the association between MetS and inflammation, which could be triggered by adiposity. In previous studies, obesity has been associated with MetS in all stages of childhood, independently of the definition used [8] and this prevalence of MetS in children and adolescents increases with the severity of obesity [9].

As adiposity is considered the common trigger for MetS and the inflammatory state, it has been suggested that both could co-exist [10], but there is some controversy [11]. The increase of the adipose tissue mass leads to an increased turnover of free fatty acids (FFAs) and changes in the secretion of pro-inflammatory adipokines [12]. This secretion of hormones and adipocytokines, such as leptin, adiponectin, resistin, and monocyte chemoattractant protein 1 (MCP-1), among others, as well as a variety of interleukins together with TNF-α, enhance the development and/or the progression of chronic diseases, including insulin resistance and chronic inflammation [13].

Puberty is an important physiological stage in childhood, characterized by changes on endocrine function, among others. It has been shown that increases in body mass index (BMI) and the propensity of overweight and obesity follow a quadratic growth curve with the steepest increase before and during puberty [14]. Additionally, some studies have shown significant differences in MetS between the prepubertal and postpubertal stages, pointing to puberty as a metabolically risky life period for children with obesity [8]. However, there is scarce information about the longitudinal association between MetS and inflammation during puberty.

Thus, the present study aimed to evaluate the association between the longitudinal trajectories of cardiometabolic and inflammatory markers during puberty and the development of MetS in a sample of Spanish children with obesity. Additionally, the risk of developing MetS during puberty was evaluated considering the prepubertal levels of assessed cardiometabolic and inflammatory biomarkers.

## Materials and methods

### Study design

For the overall PUBMEP study (“Puberty and metabolic risk in obese children. Epigenetic alterations and pathophysiological and diagnostic implications”), a total of 374 subjects were contacted, of which 49 were not located, 36 could not participate because they had changed their place of residence or met any of the exclusion criteria, and 98 declined the invitation. Out of them, 191 agreed to participate in the PUBMEP study. All these children were recruited as prepubertal children during the period 2012–2015, T1, and called again for follow-up medical consultation in 2018, T2. Children were recruited at Lozano Blesa University Clinical Hospital (Zaragoza), Santiago de Compostela University Clinical Hospital (Santiago de Compostela), and Reina Sofia University Clinical Hospital (Córdoba).

However, 75 children participated in the present study (42 boys and 33 girls) as they had complete information in both time points, T1 and T2, for all the anthropometric markers and all the cardiometabolic biomarkers.

### Ethical considerations

The PUBMEP study has been conducted following the Declaration of Helsinki (Edinburgh 2000 revised). Moreover, the study was approved by the corresponding ethic committees in each of the participating centers. All parents or guardians and children over 12 years provided written informed consent, while younger children gave their assent.

### Anthropometric measurements

A set of anthropometric markers were measured by trained researchers using standard procedures: body weight (kg, SECA 701 model class III digital display (Germany)), height (cm, a Harpenden wall-mounted stadiometer), and waist circumference (WC, SECA (Germany) (cm)) were measured twice in underwear or light sportswear and barefoot. Blood pressure was measured three times. The measurement was performed with an OMRON M3 digital blood pressure monitor (Japan) after 5 min of resting.

The BMI status (weight in kg/height in m^2^) was identified according to Cole et al. [15]. The abdominal waist measurements were related to the Spanish percentile tables of Fernández et al. [16]. Besides the assessment of the pubertal stage following the Tanner classification (1 for prepubertal and 2–5 for pubertal children) [17, 18], it was also confirmed with a hormonal study.

### Metabolic syndrome definition

To determine the presence of MS, we used the definition established by Olza et al. in 2011 [19], which includes obesity (body mass index or zBMI > 95p) and one of the following: hypertension (BP z-score > 95p), insulin resistance (HOMA IR ≥ 95p), hypertriglyceridemia (TAG z-score > 95p) [20], and low HDL-c (HDL-c < 5p) [21]. The 95th percentile of HOMA-IR was considered 2.5 in the prepubertal stage, 3.38 in pubertal boys, and 3.905 in pubertal girls established in a cohort of Spanish children [22, 23]. The values of blood pressure adjusted for sex, age, and height were classified according to international references [24].

### Biomarkers

Samples were obtained after 12 h of fasting status. Total cholesterol and triacylglycerols (TAG) (Advia 2400 Chemistry system; Siemens healthcare diagnostics, Erlangen, Germany) and HDL-c and low-density lipoprotein cholesterol were determined (LDL-c) (SAS-3 cholesterol profile kit—Helena Biosciences Europe; Tyne and Wear, UK). Plasma fasting glucose (Advia 2400 Chemistry system; Siemens healthcare diagnostics, Erlangen, Germany) and insulin (Advia centaur XP analyzer, Siemens healthcare diagnostics, Erlangen, Germany) determinations were used to calculate the HOMA-IR index.

Regarding inflammatory biomarkers, high-sensitivity C-reactive protein (hsCRP) was determined using a particle-enhanced turbidimetric immunoassay (Dade Behring Inc., Deerfield, IL, USA). The rest of the inflammatory biomarkers: adiponectin, leptin, resistin, TNF-α, IL-8, total plasminogen activator inhibitor-1 (tPAI-1), myeloperoxidase (MPO), monocyte chemoattractant protein-1 (MCP-1), soluble intercellular cell adhesion molecule-1 sICAM-1, and soluble vascular cell adhesion molecule-1 (sVCAM) were analyzed using a Luminex 200 system (Luminex Corporation, Austin, TX, USA) with human monoclonal antibodies from Millipore (EMD Millipore Corp, Billerica, MA).

### Statistical analysis

Continuous values in descriptive tables are expressed as mean and standard deviation, median and interquartile range, or sample and percentage. All variables, except age, Tanner stage, and glucose, were log-transformed. In the descriptive statistics of the sample, the overall progression of body composition and cardiometabolic and inflammatory biomarkers from prepubertal to pubertal are shown. In addition, results of the progression were analyzed by sex.

Also, a prospective analysis was performed grouping the participants by BMI category and MetS prevalence in prepuberty. Four categories were considered: normal weight (NW), overweight (OW), obesity with no-MetS (OB no-MS), and obesity with MetS (OB MS). Then, a retrospective analysis of the cardiometabolic and inflammatory biomarkers and their increment, where participants were grouped by MetS prevalence in puberty, was performed. For these analyses, mixed-effects linear models were employed. The models included fixed effects for puberty stage, group, and their interaction to allow within and between-group comparisons and Tanner stage and sex to correct for any differences in pubertal stage and sex between groups. For all the analyses, mixed-effect models were fitted for each of the variables. A random effect for participants was included to account for repeated measures.

The models were used to test for changes in outcomes during progression from prepubertal to pubertal stage within each group, pairwise outcomes differences at the prepubertal stage between groups, and pairwise differences in the prepubertal-pubertal progression between groups (Supplementary Table 1). All tests were calculated using the Kenward-Roger method for degrees of freedom and corrected for false discovery rate using the Benjamini–Yekutieli procedure.

Finally, backward stepwise model selection based on AIC was used to select the final model that best predicts MS in puberty according to prepubertal data in children with obesity in prepuberty. Variables included in the initial model were prepubertal levels of BMI-z, leptin, TNF-α, resistin, adiponectin, sICAM1, MPO, MCP1, tPAI, HOMA-IR, and age, as well as sex and Tanner stage in puberty (as confounding factors) (Supplementary Table 2).

All statistical analyses were performed using the R statistical package (4.1.0 version). Differences found were significant when *p* < 0.005.

## Results

Descriptive characteristics for the study population can be found in Table 1. Body composition and inflammatory and cardiometabolic biomarkers values in the prepubertal and pubertal stages are shown in that table for the overall population and by sex.

**Table 1:** Participant characteristics, body composition, and inflammatory markers grouped by pre and pubertal stage and by sex

	Total (***n*** = 75)			Female (***n*** = 33)			Male (***n*** = 42)		
	Pre *n* = 75	Pub *n* = 75	*p* value	Pre *N* = 33	Pub *N* = 33	*p* value	Pre *N* = 42	Pub *N* = 42	*p* value
**Age (yr)**	7.9 ± 2.1	14.6 ± 1.8	<0.001	7.4 ± 1.9	14.1 ± 1.9	<0.001	8.2 ± 2.1	15.0 ± 1.6	<0.001
**Tanner stage**	0.0 ± 0.1	4.3 ± 1.0	<0.001	0.0 ± 0.0	4.6 ± 0.9	<0.001	0.0 ± 0.0	4.0 ± 1.1	<0.001
Body composition									
**BMI (kg/m**^**2**^**)**	22.3 (7.6)	26.3 (8.5)	<0.001	20.7 (6.8)	24.8 (8.0)	0.309	23.1 (8.0)	27.0 (7.6)	<0.001
**BMI-z**	1.68 (2.85)	1.53 (2.24)	0.589	1.16 (2.3)	1.31 (2.23)	0.922	1.91 (3.20)	1.55 (2.1)	0.473
**BMI class**									
NW	23 (30.7)	24 (32.0)	0.600	11 (33.3)	13 (39.4)	0.999	12 (28.6)	11 (26.2)	0.999
OW	18 (24.0)	22 (29.3)	0.330	8 (24.2)	8 (24.2)		10 (23.8)	14 (33.3)	
OB	34 (45.3)	29 (38.7)	0.234	14 (42.4)	12 (36.4)		20 (47.6)	17 (40.5)	
**WC (cm)**	70 (21)	84 (25)	<0.001	68 (11)	79 (19)	<0.001	74 (23)	87 (27)	<0.001
**SBP (mm Hg)**	102 (16)	115 (16)	<0.001	102 (13)	112 (16)	0.999	102 (19)	117 (23)	<0.001
**DBP (mm Hg)**	62 (10)	66 (10)	0.008	62 (12)	67 (11)	0.999	62 (10)	66 (10)	0.111
**Glucose (mg/dL)**	85 ± 7	85 ± 9	0.083	85 ± 6	87 ± 9	0.053	84 ± 8	84 ± 8	0.999
**Insulin (U/L)**	6.7 (6.7)	12.3 (9.8)	<0.001	7.9 (6.6)	13.0 (9.1)	0.006	5.8 (6.2)	11.2 (8.7)	<0.001
**HOMA-IR**	1.32 (1.45)	2.57 (2.17)	<0.001	1.59 (1.4)	2.77 (1.94)	0.003	1.18 (1.36)	2.16 (1.96)	<0.001
**Cholesterol (mg/dL)**	168 (44)	153 (38)	0.005	177 (45)	152 (42)	0.999	160 (40)	155 (32)	0.048
**LDL-c (mg/dL)**	101 (38)	89 (30)	0.016	107 (39)	87 (30)	0.999	93 (29)	89 (32)	0.127
**HDL-c (mg/dL)**	54 (20)	45 (12)	<0.001	52 (22)	47 (13)	0.999	54 (12)	44 (12)	0.006
**TAG (mg/dL)**	53 33	76 (41)	<0.001	54 (26)	76 (31)	0.077	52 (34)	76 (46)	0.001
**SM no**	66 (88.0)	66 (88.0)	0.999	27 (81.8)	30 (90.9)	0.922	39 (92.9)	36 (85.7)	0.604
**SM yes**	9 (12.0)	9 (12.0)		6 (18.2)	3 (9.1)		3 (7.1)	6 (14.3)	
Inflammatory markers									
**CRP (mg/L)**	1.19 (2.35)	1.20 (3.37)	0.177	0.90 (2.30)	1.00 (3.49)	0.999	1.20 (2.75)	1.20 (3.04)	0.999
**Leptin (µg/L)**	9.77 (12.14)	9.49 (10.06)	0.725	10.13 (8.74)	12.61 (9.74)	0.044	9.19 (13.34)	6.86 (9.03)	0.998
**TNF-α (ng/L)**	2.81 (2.26)	2.41 (0.96)	0.079	2.59 (1.99)	2.33 (1.14)	0.999	3.16 (2.19)	2.45 (0.88)	0.256
**IL8 (ng/L)**	1.58 (1.19)	2.39 (1.49)	0.004	1.32 (1.10)	2.29 (1.80)	0.786	1.67 (1.14)	2.5 (1.57)	0.169
**Resistin (µg/L)**	19.57 (15.28)	19.66 (7.78)	0.956	19.31 (15.95)	21.49 (8.17)	0.999	19.77 (15.25)	18.63 (7.94)	0.999
**Adiponectin (mg/L)**	14.92 (13.43)	8.64 (8.95)	<0.001	16.43 (13.19)	11.94 (11.93)	0.366	14.00 (9.2)	6.64 (6.34)	<0.001
**MPO (µg/L)**	23.26 (38.49)	23.50 (60.74)	0.494	28.85 (39.56)	18.87 (34.6)	0.999	18.9 (31.07)	32.38 (108.72)	0.169
**MCP1 (ng/L)**	97.45 (55.02)	110.74 (41.02)	0.180	97.45 (65.79)	105.26 (31.09)	0.999	97.02 (44.01)	120.12 (58.46)	0.588
**tPAI (µg/L)**	19.17 (17.81)	13.67 (14.81)	0.052	19.17 (17.02)	13.22 (10.91)	0.922	17.99 (18.61)	13.75 (16.44)	0.651
**sICAM1 (mg/L)**	0.11 (0.09)	0.08 (0.03)	0.057	0.12 (0.07)	0.08 (0.03)	0.999	0.09 (0.11)	0.09 (0.09)	0.048

Statistics presented: mean ± SD; *n* (%), median (interquartile range). Pre-pub difference in total is controlled for sex and Tanner stage. Pre-pub differences and differences between sexes (in text) are controlled for Tanner stage. *p* values are adjusted for false discovery rate using Benjamini–Yekutieli procedure

*BMI* body mass index, *NW* normal weight, *OW* overweight, *OB* obesity, *WC* waist circumference, *SBP* systolic blood pressure, *DBP* diastolic blood pressure, *HOMA-IR* homeostasis model assessment for insulin resistance, *LDL-c* low-density lipoproteins-cholesterol, *HDL-c* high-density lipoproteins-cholesterol, *TAG* triglycerides, *CRP* C-reactive protein, *TNF-α* tumoral necrosis factor-alpha, *IL8* interleukin 8, *MPO* myeloperoxidase, *MCP1* monocyte chemoattractant protein-1, *tPAI* total plasminogen activator inhibitor, *sICAM* soluble intercellular adhesion molecule-1

There were differences by pubertal stage in the overall population, being the concentrations of those biomarkers included in the MetS definition, DBP, SBP, HOMA-IR, and TAG, significantly higher in puberty (*p* < 0.05). On the other hand, HDL-c and LDL-c had significantly lower concentrations in puberty when compared with prepuberty (*p* < 0.005). Regarding the inflammatory biomarkers, IL8 and adiponectin showed significant differences by pubertal stage (*p* < 0.05). For girls, significant differences (*p* < 0.05) by pubertal status were found for HOMA-IR, among the MetS components, and leptin, while, for boys, those differences (*p* < 0.05) were found for SBP, HOMA-IR, TAG, and HDL-c and, among the inflammatory biomarkers, for adiponectin (*p* < 0.001).

In Table 2, participants are divided by BMI category and prevalence of MetS in prepuberty (NW, OW, OB no-MS, and OB MS) and their body composition markers, cardiometabolic and inflammatory variables were assessed in the prepubertal, the pubertal stage and the increase or change, i.e., T2 values − T1 values. Regarding the within-group changes, age and WC were the variables that showed significant differences from pre to pubertal stage within all assessed groups: NW, OW, OB no-MS, and OB MS. Prepubertal differences between groups showed that NW subjects had significantly lower values than the rest of the groups for BMI-z, WC, insulin, HOMA-IR, and leptin. Those in the prepubertal NW groups had also lower values of tPAI in comparison with those in the OB groups: OB no-MS and OB MS.

**Table 2:** Pre and pubertal levels and change of body composition and inflammatory markers, grouped by BMI class in prepuberty

Variable	Normal weight in prepuberty (*n* = 23)			Overweight in prepuberty (*n* = 18)			Obesity no-MS in prepuberty (*n* = 25)			Obesity MS in prepuberty (*n* = 9)		
	Pre	Pub	Increment	Pre	Pub	Increment	Pre	Pub	Increment	Pre	Pub	Increment
**Age (years)**	7.9 ± 2.1	14.8 ± 1.7	6.9 ± 3.1*	8.7 ± 1.9^c^	14.6 ± 2.1	5.9 ± 2.7*	7.2 ± 2.2^b^	14.3 ± 1.8	7.1 ± 2.8*	7.8 ± 1.6	14.6 ± 1.4	6.8 ± 2.2*
**Tanner stage**	0.0 ± 0.0	4.3 ± 1.1	4.3 ± 1.1	0.0 ± 0.0	14.6 ± 2.1	4.4 ± 0.9	0.0 ± 0.0	4.1 ± 1.1	4.1 ± 1.1	0.0 ± 0.0	4.6 ± 0.9	4.6 ± 0.9
**BMI (kg/m**^**2**^**)**	16.2 (1.5)	20.7 (3.3)	3.7 (3.3)	20.9 (2.9)	26.5 (3.0)	5.4 (4.4)	25.1 (3.1)	29.8 (8.4)	4.5 (5.8)	24.5 (4.8)	33.3 (8.3)	7.2 (6.5)
**BMI-z**	−0.36 (0.84)^bcd^	−0.02 (0.56)	0.11 (0.80)^e^	1.37 (0.61)^acd^	1.46 (1.15)	0.01 (1.08)^e^	3.56 (2.17)^ab^	2.53 (1.60)	−1.11 (2.25)*^e^	3.01 (1.21)^ab^	3.45 (2.31)	0.14 (1.33)
**WC (cm)**	56 (4)^bcd^	70 (8)	12 (10)*	70 (12)^acd^	84 (13)	14 (17)*	78 (14)^ab^	99 (22)	16 (15)*	84 (16)^ab^	106 (25)	17 (10)*
**SBP (mm Hg)**	100 (12)^d^	105 (15)	4 (20)^e^	104 (14)	114 (22)	10 (12)	100 (15)^d^	120 (17)	20 (17)*^e^	117 (9)^ac^	118 (11)	1 (15)
**DBP (mm Hg)**	62 (4)^d^	64 (10)	3 (12)	57 (12)^d^	68 (6)	9 (7)*	64 (11)^d^	67 (14)	7 (18)*	78 (10)^abc^	77 (25)	−4 (26)
**Glucose (mg/dL)**	84 ± 7	86 ± 9	2 ± 11	86 ± 6	87 ± 6	1 ± 7	82 ± 6	83 ± 9	1 ± 10	88 ± 8	85 ± 10	−3 ± 12
**Insulin (U/L)**	5.0 (5.1)^bcd^	9.9 (8.8)	5.5 (7.5)*	6.8 (7.5)^a^	11.1 (10.9)	3.2 (5.4)	7.9 (6.9) ^a^	12.3 (6.1)	5.1 (9.7)*	11.0 (7.2)^a^	17.9 (12.0)	5.3 (14.7)
**HOMA-IR**	1.05 (0.99)^bcd^	2.23 (1.67)	1.49 (1.36)*	1.41 (1.58)^a^	2.47 (2.22)	0.75 (1.08)	1.36 (1.56)^a^	2.52 (1.45)	1.03 (2.17)	2.17 (1.58)^a^	4.55 (2.29)	1.07 (2.88)
**Cholesterol (mg/dL)**	161 (50)	151 (32)	−13 (26)	160 (24)	146 (42)	−26 (27)	173 (45)	160 (32)	−9 (30)	185 (47)	171 (44)	−20 (15)
**LDL-c (mg/dL)**	90 (36)	86 (21)	−3 (29)	95 (33)	87 (26)	−13 (13)	105 (30)	96 (28)	−7 (21)	107 (40)	112 (35)	−21 (16)
**HDL-c (mg/dL)**	59 (26)^d^	52 (20)	−5 (18)	60 (20)^d^	44 (9)	−12 (13)*	51 (15)	45 (8)	−5 (9)	42 (8)^ab^	40 (10)	−4 (8)
**TAG (mg/dL)**	52 (16)^d^	66 (28)	19 (28)*	59 (27)	76 (37)	11 (30)	55 (31)	78 (55)	18 (54)*	101 (22)^a^	110 (55)	10 (73)
**CRP (mg/L)**	0.30 (0.80)^c^	0.70 (1.23)	0.13 (1.15)	1.30 (1.65)	1.58 (3.94)	0.47 (2.62)	2.10 (2.40)^a^	1.20 (5.16)	0.50 (4.04)	0.90 (2.00)	1.90 (9.53)	1.00 (5.90)
**Leptin (µg/L)**	1.76 (2.11)^bcd^	4.49 (7.01)	1.40 (4.45)*	10.02 (4.09)^a^	11.73 (6.78)	1.89 (7.38)	13.30 (12.44)^a^	10.99 (11.49)	2.47 (16.03)	21.71 (16.74)^a^	17.14 (6.60)	−8.15 (15.44)
**TNF-α (ng/L)**	2.81 (2.12)	2.41 (0.82)	−0.38 (2.26)	1.81 (1.93)^c,d^	2.46 (1.02)	0.51 (1.60)^e^	3.45 (1.59)^b^	2.34 (0.50)	−1.09 (1.39)*^e^	3.06 (2.79)^b^	2.91 (1.24)	−0.91 (1.86)
**IL8 (ng/L)**	1.46 (1.27)	1.98 (1.65)	0.73 (2.07)	1.23 (1.77)	2.62 (1.84)	1.06 (1.95)	1.58 (0.98)	2.51 (1.35)	0.87 (1.82)	1.80 (0.58)	2.33 (1.33)	0.39 (1.02)
**Resistin (µg/L)**	21.99 (20.39)	16.99 (7.09)	−3.15 (13.08)	19.23 (15.18)	20.37 (9.76)	−1.92 (11.64)	16.27 (15.58)	21.00 (4.51)	5.49 (16.78)*	21.29 (8.68)	21.88 (8.49)	3.06 (8.39)
**Adiponectin (mg/L)**	16.59 (14.73)	11.10 (11.07)	−5.30 (13.69)*	12.61 (8.80)	6.28 (5.22)	−6.28 (8.06)*	13.97 (10.95)	8.54 (7.21)	−4.60 (9.66)*	15.05 (10.44)	9.05 (6.99)	−4.21 (12.80)
**MPO (µg/L)**	17.40 (35.71)	16.80 (22.52)	−4.88 (40.43)	39.97 (55.94)	22.59 (100.08)	1.63 (164.78)	17.40 (29.60)	43.26 (90.52)	15.23 (106.01)	30.54 (16.36)	22.38 (25.76)	−11.93 (56.21)
**MCP1 (ng/L)**	83.92 (39.96)	112.61 (32.55)	31.77 (50.25)	78.92 (47.33)	97.44 (53.29)	16.65 (56.84)	104.19 (48.26)	114.70 (36.65)	3.25 (42.71)	127.75 (44.32)	107.13 (52.62)	−9.82 (45.99)
**tPAI (µg/L)**	11.35 (12.90)^cd^	10.24 (6.06)	−1.52 (14.20)	14.55 (18.22)	13.53 (8.27)	−2.79 (13.17)	19.69 (15.36)^a^	21.81 (17.55)	−5.47 (22.32)	31.73 (10.54)^a^	20.20 (12.36)	−12.73 (9.27)
**sICAM1 (mg/L)**	0.11 (0.09)	0.07 (0.03)	−0.01 (0.08)	0.09 (0.06)	0.09 (0.03)	−0.01 (0.03)	0.11 (0.10)	0.09 (0.04)	0.01 (0.14)	0.11 (0.05)	0.09 (0.02)	−0.01 (0.06)

Statistics presented: mean ± SD; *n* (%), median (interquartile range). Pre-pub evolution, group differences in prepuberty and in evolution (in text) were controlled for sex and Tanner stage. *p* values are adjusted for false discovery rate using Benjamini–Yekutieli procedure

*BMI* body mass index, *NW* normal weight, *OW* overweight, *OB* obesity, *WC* waist circumference, *SBP* systolic blood pressure, *DBP* diastolic blood pressure, *HOMA-IR* homeostasis model assessment for insulin resistance, *LDL-c* low-density lipoproteins-cholesterol, *HDL-c* high-density lipoproteins-cholesterol, *TAG* triglycerides, *CRP* C-reactive protein, *TNF-α* tumoral necrosis factor-alpha, *IL8* interleukin 8, *MPO* myeloperoxidase, *MCP1* monocyte chemoattractant protein-1, *tPAI* total plasminogen activator inhibitor, *sICAM* soluble intercellular adhesion molecule-1

^*^Significant change from pre to pubertal stage within group

^a^significant difference from normal weight

^b^significant difference from overweight

^c^significant difference from obesity no-MS

^d^significant difference from obesity MS

^e^Increment differences between evolution groups (*p* < 0.005)

To analyze how prepubertal values of cardiometabolic and inflammatory biomarkers, and their increment, are associated with the prevalence of MetS in puberty, we post-categorized the subjects based on if they presented MS in puberty or not. Descriptive statistics of participants at the prepubertal and pubertal stage, as well as the prepubertal-pubertal increment, are presented in Table 3, grouped by MetS prevalence in puberty. Significant changes (*p* < 0.05) from pre- to pubertal stages were found for BMI, WC, insulin, HOMA-IR, cholesterol, HDL-c, TAG, IL-8, and adiponectin within the group of those no-MS in puberty. The prepubertal values of BMI-z, WC, HOMA-IR, leptin, and tPAI were higher between groups for those that developed MS in puberty. For those with MetS in puberty, significant increments from pre- to pubertal stages were found for BMI, WC, and DBP. There were no significant between-group differences in the evolution of the anthropometry and cardiometabolic and inflammatory biomarkers between participants with and without MS in puberty (data not shown).

**Table 3:** Pre and pubertal levels and change of body composition and cardiometabolic risk markers grouped by the presence or absence of metabolic syndrome in puberty

Variable	No MS in puberty (*n* = 66)			MS in puberty (*n* = 9)			*p* value
	Pre	Pub	Increment	Pre	Pub	Increment	
**Age (years)**	7.8 ± 2.1	14.6 ± 1.8	6.7 ± 2.8*	7.9 ± 2.4	14.4 ± 1.6	6.5 ± 2.7*	0.999
**Tanner stage**	0.0 ± 0.0	4.3 ± 1.0	4.3 ± 1.0*	0.0 ± 0.0	4.3 ± 1.0	4.3 ± 1.0*	0.999
**BMI (kg/m**^**2**^**)**	21.0 (7.1)	25.2 (7.1)	3.9 (4.8)*	26.8 (3.2)	34.9 (8.0)	8.0 (4.4)*	0.009
**BMI-z**	1.41 (2.69)	1.28 (2.08)	−0.04 (1.12)	3.83 (3.45)	3.20 (2.40)	0.10 (0.96)	0.010
**WC (cm)**	68 (19)	80 (20)	14 (15)*	82 (13)	110 (10)	23 (4)*	0.028
**SBP (mm Hg)**	102 (14)	113 (15)	9 (20)	110 (17)	125 (17)	25 (21)	0.999
**DBP (mm Hg)**	62 (10)	65 (8)	5 (14)	64 (13)	82 (10)	18 (9)*	0.999
**Glucose (mg/dL)**	84 ± 7	85 ± 8	1 ± 9	90 ± 7	88 ± 10	−1 ± 15	0.290
**Insulin (U/L)**	6.4 (5.1)	11.1 (8.5)	4.3 (8.2)*	14.0 (7.1)	20.9 (11.9)	9.8 (6.2)	0.010
**HOMA-IR**	1.28 (1.17)	2.28 (1.82)	0.93 (1.46)*	3.00 (2.28)	3.92 (3.23)	1.48 (2.11)	0.009
**Cholesterol (mg/dL)**	167 (43)	152 (40)	−18 (32)*	180 (47)	160 (32)	−20 (22)	0.999
**LDL-c (mg/dL)**	101 (32)	88 (28)	−11 (26)	120 (37)	107 (37)	−13 (15)	0.999
**HDL-c (mg/dL)**	55 (18)	48 (13)	−7 (15)*	43 (7)	41 (6)	−7 (4)	0.087
**TAG (mg/dL)**	52 (21)	72 (37)	13 (36)*	99 (25)	111 (40)	24 (29)	0.153
**Inflammatory biomarkers**							
**CRP (mg/L)**	1.04 (2.47)	1.20 (3.29)	0.42 (2.79)	1.20 (1.13)	1.15 (5.60)	0.49 (5.55)	0.999
**Leptin (µg/L)**	8.48 (9.63)	9.25 (9.69)	1.35 (7.22)	21.42 (10.49)	19.69 (15.34)	−7.41 (13.25)	0.002
**TNF-α (ng/L)**	2.86 (2.42)	2.41 (0.88)	−0.62 (2.04)	2.77 (1.84)	2.45 (0.97)	−0.73 (1.19)	0.999
**IL8 (ng/L)**	1.60 (1.19)	2.51 (1.65)	0.89 (2.11)*	1.35 (0.65)	2.12 (0.56)	0.39 (1.55)	0.999
**Resistin (µg/L)**	19.23 (16.33)	19.64 (7.66)	1.05 (11.52)	21.29 (17.09)	21.00 (6.97)	−2.83 (18.86)	0.999
**Adiponectin (mg/L)**	14.09 (10.76)	8.84 (8.82)	−5.62 (12.18)*	17.75 (14.78)	8.54 (5.15)	−6.99 (8.59)	0.999
**MPO (µg/L)**	23.24 (38.78)	23.43 (69.28)	1.94 (81.86)	28.85 (18.44)	31.97 (38.70)	4.25 (55.33)	0.999
**MCP1 (ng/L)**	91.75 (56.80)	109.38 (38.23)	8.01 (54.31)	117.09 (23.56)	114.70 (70.10)	17.78 (61.32)	0.999
**tPAI (µg/L)**	16.30 (14.26)	13.19 (14.82)	−3.07 (19.49)	37.80 (23.83)	20.20 (17.09)	−10.98 (16.58)	0.010
**sICAM1 (mg/L)**	0.11 (0.10)	0.08 (0.03)	−0.01 (0.08)	0.10 (0.05)	0.09 (0.24)	0.02 (0.21)	0.031

Statistics presented: mean ± SD; *n* (%), median (interquartile range). Pre-pub evolution, group differences in prepuberty and in evolution (in text) were controlled for sex and Tanner stage. *p* values are adjusted for false discovery rate using Benjamini–Yekutieli procedure

*BMI* body mass index, *NW* normal weight, *OW* overweight, *OB* obesity, *WC* waist circumference, *SBP* systolic blood pressure, *DBP* diastolic blood pressure, *HOMA-IR* homeostasis model assessment for insulin resistance, *LDL-c* low-density lipoproteins-cholesterol, *HDL-c* high-density lipoproteins-cholesterol, *TAG* triglycerides, *CRP* C-reactive protein, *TNF-α* tumoral necrosis factor-alpha, *IL8* interleukin 8, *MPO* myeloperoxidase, *MCP1* monocyte chemoattractant protein-1, *tPAI* total plasminogen activator inhibitor, *sICAM* soluble intercellular adhesion molecule-1

^*^Significant change from pre to pubertal stage within group

As all participants who presented MetS in puberty were obese, in Table 4 results of a stepwise logistic regression to test which prepubertal inflammatory biomarker helps the most to predict MetS are shown. For this, we restricted analyses to the group of 34 children with obesity in the prepubertal stage. Within these participants, among the inflammatory biomarkers included in the model, tPAI significantly (*p* < 0.017) helped to predict MetS in puberty (OR = 1.19; CI: 1.06–1.43).

**Table 4:** Logistic model for predicting prevalence of metabolic syndrome in puberty for participants with obesity in prepuberty

*Predictors*	*Metabolic syndrome in puberty*				
	*OR*	*OR 95% CI*	*β*	*β 95% CI*	*p*
(Intercept)	0.00	0.00–0.00	0.14	0.02–0.49	0.020
Age prepubertal	4.37	1.34–24.44	20.70	1.82–711.28	0.037
Tanner prepubertal	2.71	0.72–14.66	2.82	0.71–16.24	0.176
BMI-z prepubertal	4.27	1.39–22.59	13.56	1.81–271.04	0.034
Leptin prepubertal	0.83	0.64–0.98	0.11	0.01–0.84	0.062
tPAI prepubertal	1.19	1.06–1.43	10.58	2.10–118.15	0.017
HOMA-IR prepubertal	2.28	1.02–6.35	3.95	1.03–21.89	0.059

Backwards stepwise model selection based on AIC was used to select the final model that best predict MS in puberty for participants with obesity in prepuberty. Variables included in the initial model were prepubertal levels of BMI-z, leptin, TNF-α, resistin, adiponectin, sICAM1, MPO, MCP1, tPAI, HOMA-IR, and age, as well as sex and Tanner stage in puberty

*CI* confidence interval, *BMI-z* BMI z-score, *tPAI* tissue plasminogen activator inhibitor, *HOMA-IR* homeostatic model assessment of insulin resistance

## Discussion

In this study, significant differences in obesity degree and MetS were observed longitudinally for the mean concentrations of a set of anthropometric and cardiovascular/inflammatory biomarkers in childhood. Those with MetS in puberty had already higher values of several cardiometabolic biomarkers in prepuberty. Finally, it was observed that the odds of having MetS in puberty were significantly higher when having high zBMI or high concentrations of tPAI, for those obese in prepuberty.

Puberty is a transition period, characterized by physiologic change, including secretion of sex steroids, acceleration in growth, and accumulation of both lean and fat mass. Puberty is also a period of change for the cardiometabolic risk factors, insulin resistance, plasma lipids, blood pressure, and adipokines [25–27]. Also, puberty is one of the greatest risk factors for impairment of body composition [28] and the transition from metabolically healthy to unhealthy obesity [29].

Overall, in our sample, we observed a derangement associated with pubertal development for most of the cardiometabolic markers in both sexes. However, for cholesterol and LDL-c we found a better profile in those pubertal when compared with the prepubertal participants. Previous evidence suggests that there are changes in total cholesterol and lipid profiles during puberty. Out of these changes, the most well-described are the decrease in total and low-density lipoprotein cholesterol during puberty which are in line with our results [26, 30–32]. It has been suggested that the changes in lipid levels occurring during adolescence and sexual maturation are closely related to the alterations in hormones during this period of life. However, most of the studies assessing changes in lipids over time do not consider pubertal maturation so results are mostly based on cross-sectional studies.

Research on the effects of hormones on immune functioning also suggests a link between pubertal development and inflammatory physiology [33]. Prior works suggest that sex hormones, which increase during puberty, can have an immunomodulatory effect on inflammatory biomarkers [27]. In our sample, we found an increase of sICAM in pubertal boys. Also, changes in adiponectin and leptin could occur during puberty. Leptin plays a role in the initiation of puberty and increases in puberty [34]. However, sex differences could be found. In girls, leptin increases throughout puberty to adulthood, whereas in boys, leptin tends to decrease [35, 36]. In the present study, leptin was significantly increased in pubertal girls while for pubertal boys the value was lower than in prepuberty even if the decrease was not significant. In adults, sex is the major determinant of leptin concentrations, being higher in women than men [37]. In our study, adiponectin changes differentially in boys’ and girls’ puberty: in girls, there could be a small change, whereas in boys decreases at pubertal onset and continues to go down as puberty progresses [38, 39]. This is in line with our results, where a significant decrease in adiponectin was found in pubertal boys.

In our sample, 12% of the participants exhibited features of MetS. However, there is no consensus regarding the definition of the MetS for children, which makes more difficult the diagnosis [40]. Depending on the definition used, prevalence ranged from 7.6 to 30.8% in a sample of Spanish children [41]. Despite this, it seems clear that MetS is linked to several cardiometabolic diseases and that could increase the risk of future comorbidities. However, only a few studies have examined the link between early life MetS and future cardiovascular disease [6, 42]. Results from a longitudinal study showed that MetS in childhood, combined with changes in age-specific BMI percentile, predicted cardiovascular events later in adulthood [6]. In addition, a study from Magnussen et al. [42] found that MetS in youth could predict MetS in adulthood and high cIMT and T2DM in early to middle adulthood.

In the retrospective analysis conducted comparing the cardiometabolic and inflammatory biomarkers during puberty by prepubertal obesity degree and the prepubertal presence or not of MetS, we observed that children with obesity but no MetS at prepuberty had significantly lower values of zBMI, TNF, resistin, and adiponectin between the pubertal stages. In literature, resistin has been shown to be a potential activator of inflammatory markers and expression of cellular adhesion molecules (CAMs) on the endothelial surface [43]. It has been observed that adolescents with obesity have higher values of resistin independently of their adipose tissue [44]. However, it seems to be an association between resistin, gender, and Tanner stage, which has also been found for adiponectin, that could affect the concentrations [39, 45].

In the longitudinal analysis, we found differences by pubertal status on the cardiometabolic and inflammatory concentrations by MetS status in puberty. Those with MetS in puberty had significantly higher prepubertal values of BMI, zBMI, WC, insulin, HOMA-IR, leptin, and t-PAI than those without MetS. These results suggest that there are already cardiometabolic and inflammatory biomarkers increased during prepuberty for those that will present MetS in the future. Leptin and tPAI are adipokines that have been involved with MetS and the inflammatory process [46]. Also, in previous studies, tPAI has been increased in children with obesity and MetS [22, 47] and also in metabolically unhealthy children [23]. In this last study, PAI-1 was increased in metabolically unhealthy children with overweight/obesity in the prepubertal stage.

In line with these results, we also investigated if having higher prepubertal values of specific biomarkers when obese could predict the prevalence of MetS in puberty. Out of all the cardiometabolic biomarkers investigated, prepubertal tPAI showed associations with future MetS. Possible mechanisms linking increased circulating PAI-1 levels to the MetS suggest that tPAI could have a significant role in metabolic health [48]. Out of the mechanisms, tPAI contributes to the development of adipose tissue and insulin resistance [48]. However, the mechanisms seem complex and probably are interrelated so more longitudinal studies are needed to investigate in deep these associations. In a previous cross-sectional study in adults, PAI-1 and adiponectin showed the most robust associations with MetS components in a general population, indicating that unfavorable adipose tissue performance is a key contributor to these metabolic anomalies [49].

The present work has several limitations. The sample size was low which could affect to the statistical power and to enroll more population would be needed to validate the higher tPAI odds with biomarkers. Also, the analysis was not stratified by sex, due to the sample size. As strengths, we managed to get complete data on the prepubertal and pubertal states of children from three Spanish regions. Also, this is one of the first studies to analyze the association between MetS and cardiometabolic inflammatory biomarkers, assessing the association in both directions, in a sample of prepubertal and pubertal children. Finally, children were individually examined by clinicians to determine their Tanner stage and the diagnosis was confirmed with hormonal variables.

In conclusion, those with MetS in puberty had already higher values of several cardiometabolic biomarkers in prepuberty suggesting that prepuberty is a critical period for the onset of future MetS. In line with this, those with higher prepubertal tPAI had 19% odds of having MetS at puberty which highlights the bi-directional association between MetS and specific biomarkers. Thus, assessing cardiometabolic status already at prepuberty is key to avoiding future comorbidities. Further studies with large population are needed to confirm these results.

## Supplementary Information

Below is the link to the electronic supplementary material.

Supplementary file1 (DOCX 103 kb)
