# Supplementary material for: Longitudinal associations between cardiovascular biomarkers and metabolic syndrome during puberty: the PUBMEP study
Source: Eur J Pediatr. 2022 Nov 15;182(1):419–29. doi: 10.1007/s00431-022-04702-6 (PMC9829643; doi:10.1007/s00431-022-04702-6)
Supplement: Supplementary file 1 — Supplementary file1 (DOCX 103 kb) [file 431_2022_4702_MOESM1_ESM.docx]

**Table 1**. Participant characteristics, body composition and inflammatory markers grouped by pre and pubertal stage and by sex.

|  | Total (n = 75) | | | Female (n = 33) | | | Male (n = 42) | | |
| --- | --- | --- | --- | --- | --- | --- | --- | --- | --- |
|  | Pre  n=75 | Pub  n=75 | p-value | Pre  N=33 | Pub  N=33 | p-value | Pre  N=42 | Pub  N=42 | p-value |
| Age (yr) | 7.9 ± 2.1 | 14.6 ± 1.8 | <0.001 | 7.4 ± 1.9 | 14.1 ± 1.9 | <0.001 | 8.2 ± 2.1 | 15.0 ± 1.6 | <0.001 |
| Tanner stage | 0.0 ± 0.1 | 4.3 ± 1.0 | <0.001 | 0.0 ± 0.0 | 4.6 ± 0.9 | <0.001 | 0.0 ± 0.0 | 4.0 ± 1.1 | <0.001 |
| Body composition | | | | | | | | | |
| BMI (kg/m^2^) | 22.3 (7.6) | 26.3 (8.5) | <0.001 | 20.7 (6.8) | 24.8 (8.0) | 0.309 | 23.1 (8.0) | 27.0 (7.6) | <0.001 |
| BMI-z | 1.68 (2.85) | 1.53 (2.24) | 0.589 | 1.16 (2.3) | 1.31 (2.23) | 0.922 | 1.91 (3.20) | 1.55 (2.1) | 0.473 |
| BMI class |  |  |  |  |  |  |  |  |  |
| NW | 23 (30.7) | 24 (32.0) | 0.600 | 11 (33.3) | 13 (39.4) | 0.999 | 12 (28.6) | 11 (26.2) | 0.999 |
| OW | 18 (24.0) | 22 (29.3) | 0.330 | 8 (24.2) | 8 (24.2) |  | 10 (23.8) | 14 (33.3) |  |
| OB | 34 (45.3) | 29 (38.7) | 0.234 | 14 (42.4) | 12 (36.4) |  | 20 (47.6) | 17 (40.5) |  |
| WC (cm) | 70 (21) | 84 (25) | <0.001 | 68 (11) | 79 (19) | <0.001 | 74 (23) | 87 (27) | <0.001 |
| SBP (mm Hg) | 102 (16) | 115 (16) | <0.001 | 102 (13) | 112 (16) | 0.999 | 102 (19) | 117 (23) | <0.001 |
| DBP (mm Hg) | 62 (10) | 66 (10) | 0.008 | 62 (12) | 67 (11) | 0.999 | 62 (10) | 66 (10) | 0.111 |
| Glucose (mg/dL) | 85 ± 7 | 85 ± 9 | 0.083 | 85 ± 6 | 87 ± 9 | 0.053 | 84 ± 8 | 84 ± 8 | 0.999 |
| Insulin (U/L) | 6.7 (6.7) | 12.3 (9.8) | <0.001 | 7.9 (6.6) | 13.0 (9.1) | 0.006 | 5.8 (6.2) | 11.2 (8.7) | <0.001 |
| HOMA-IR | 1.32 (1.45) | 2.57 (2.17) | <0.001 | 1.59 (1.4) | 2.77 (1.94) | 0.003 | 1.18 (1.36) | 2.16 (1.96) | <0.001 |
| Cholesterol (mg/dL) | 168 (44) | 153 (38) | 0.005 | 177 (45) | 152 (42) | 0.999 | 160 (40) | 155 (32) | 0.048 |
| LDL-c (mg/dL) | 101 (38) | 89 (30) | 0.016 | 107 (39) | 87 (30) | 0.999 | 93 (29) | 89 (32) | 0.127 |
| HDL-c (mg/dL) | 54 (20) | 45 (12) | <0.001 | 52 (22) | 47 (13) | 0.999 | 54 (12) | 44 (12) | 0.006 |
| TAG (mg/dL) | 53 33 | 76 (41) | <0.001 | 54 (26) | 76 (31) | 0.077 | 52 (34) | 76 (46) | 0.001 |
| SM no | 66 (88.0) | 66 (88.0) | 0.999 | 27 (81.8) | 30 (90.9) | 0.922 | 39 (92.9) | 36 (85.7) | 0.604 |
| SM yes | 9 (12.0) | 9 (12.0) |  | 6 (18.2) | 3 (9.1) |  | 3 (7.1) | 6 (14.3) |  |
| Inflammatory markers | | | | | | | | | |
| CRP (mg/L) | 1.19 (2.35) | 1.20 (3.37) | 0.177 | 0.90 (2.30) | 1.00 (3.49) | 0.999 | 1.20 (2.75) | 1.20 (3.04) | 0.999 |
| Leptin (ug/L) | 9.77 (12.14) | 9.49 (10.06) | 0.725 | 10.13 (8.74) | 12.61 (9.74) | 0.044 | 9.19 (13.34) | 6.86 (9.03) | 0.998 |
| TNF-α (ng/L) | 2.81 (2.26) | 2.41 (0.96) | 0.079 | 2.59 (1.99) | 2.33 (1.14) | 0.999 | 3.16 (2.19) | 2.45 (0.88) | 0.256 |
| IL8 (ng/L) | 1.58 (1.19) | 2.39 (1.49) | 0.004 | 1.32 (1.10) | 2.29 (1.80) | 0.786 | 1.67 (1.14) | 2.5 (1.57) | 0.169 |
| Resistin (ug/L) | 19.57 (15.28) | 19.66 (7.78) | 0.956 | 19.31 (15.95) | 21.49 (8.17) | 0.999 | 19.77 (15.25) | 18.63 (7.94) | 0.999 |
| Adiponectin (mg/L) | 14.92 (13.43) | 8.64 (8.95) | <0.001 | 16.43 (13.19) | 11.94 (11.93) | 0.366 | 14.00 (9.2) | 6.64 (6.34) | <0.001 |
| MPO (ug/L) | 23.26 (38.49) | 23.50 (60.74) | 0.494 | 28.85 (39.56) | 18.87 (34.6) | 0.999 | 18.9 (31.07) | 32.38 (108.72) | 0.169 |
| MCP1 (ng/L) | 97.45 (55.02) | 110.74 (41.02) | 0.180 0.893 | 97.45 (65.79) | 105.26 (31.09) | 0.999 | 97.02 (44.01) | 120.12 (58.46) | 0.588 |
| tPAI (ug/L) | 19.17 (17.81) | 13.67 (14.81) | 0.052 0.239 | 19.17 (17.02) | 13.22 (10.91) | 0.922 | 17.99 (18.61) | 13.75 (16.44) | 0.651 |
| sICAM1 (mg/L) | 0.11 (0.09) | 0.08 (0.03) | 0.057 | 0.12 (0.07) | 0.08 (0.03) | 0.999 | 0.09 (0.11) | 0.09 (0.09) | 0.048 |

Statistics presented: Mean ± SD; n (%), median (interquartile range). Pre-pub difference in total are controlled for sex and Tanner stage. Pre-pub differences and differences between sexes (in text) are controlled for Tanner stage. P-values are adjusted for false discovery rate using Benjamini–Yekutieli procedure.

*Abbreviations: BMI, body mass index; NW, normal weight, OW, overweight; OB, obesity; WC, waist circumference; SBP, systolic blood pressure; DBP, diastolic blood pressure; HOMA-IR, homeostasis model assessment for insulin resistance; LDL-c, low-density lipoproteins-cholesterol; HDL-c, high-density lipoproteins-cholesterol; TAG, triglycerides; CRP, C-reactive protein; TNF-α: tumoral necrosis factor-alpha; IL8: interleukin 8; MPO: myeloperoxidase; MCP1: Monocyte chemoattractant protein-1; tPAI: total plasminogen activator inhibitor; sICAM: soluble intercellular adhesion molecule-1.*

**Table 2**. Pre and pubertal levels and change of body composition and inflammatory markers, grouped by BMI class in prepuberty

|  | Normal weight in prepuberty (n = 23) | | | Overweight in prepuberty (n = 18) | | | Obesity No-MS in prepuberty (n = 25) | | | Obesity MS in prepuberty (n = 9) | | |
| --- | --- | --- | --- | --- | --- | --- | --- | --- | --- | --- | --- | --- |
| Variable | Pre | Pub | Increment | Pre | Pub | Increment | Pre | Pub | Increment | Pre | Pub | Increment |
| **Age (years)** | 7.9 ± 2.1 | 14.8 ± 1.7 | 6.9 ± 3.1* | 8.7 ± 1.9^c^ | 14.6 ± 2.1 | 5.9 ± 2.7* | 7.2 ± 2.2^b^ | 14.3 ± 1.8 | 7.1 ± 2.8* | 7.8 ± 1.6 | 14.6 ± 1.4 | 6.8 ± 2.2* |
| **Tanner stage** | 0.0 ± 0.0 | 4.3 ± 1.1 | 4.3 ± 1.1 | 0.0 ± 0.0 | 14.6 ± 2.1 | 4.4 ± 0.9 | 0.0 ± 0.0 | 4.1 ± 1.1 | 4.1 ± 1.1 | 0.0 ± 0.0 | 4.6 ± 0.9 | 4.6 ± 0.9 |
| **BMI (kg/m2)** | 16.2 (1.5) | 20.7 (3.3) | 3.7 (3.3) | 20.9 (2.9) | 26.5 (3.0) | 5.4 (4.4) | 25.1 (3.1) | 29.8 (8.4) | 4.5 (5.8) | 24.5 (4.8) | 33.3 (8.3) | 7.2 (6.5) |
| **BMI-z** | -0.36 (0.84)^bcd^ | -0.02 (0.56) | 0.11(0.80)† | 1.37 (0.61)^acd^ | 1.46 (1.15) | 0.01 (1.08)† | 3.56 (2.17)^ab^ | 2.53 (1.60) | -1.11 (2.25)*† | 3.01 (1.21)^ab^ | 3.45 (2.31) | 0.14 (1.33) |
| **WC (cm)** | 56 (4)^bcd^ | 70 (8) | 12 (10)* | 70 (12)^acd^ | 84 (13) | 14 (17)* | 78 (14)^ab^ | 99 (22) | 16 (15)* | 84 (16)^ab^ | 106 (25) | 17 (10)* |
| **SBP (mm Hg)** | 100 (12)^d^ | 105 (15) | 4 (20)† | 104 (14) | 114 (22) | 10 (12) | 100 (15)^d^ | 120 (17) | 20 (17)*† | 117 (9)^ac^ | 118 (11) | 1 (15) |
| **DBP (mm Hg)** | 62 (4)^d^ | 64 (10) | 3 (12) | 57 (12)^d^ | 68 (6) | 9 (7)* | 64 (11)^d^ | 67 (14) | 7 (18)* | 78 (10)^abc^ | 77 (25) | -4 (26) |
| **Glucose (mg/dL)** | 84 ± 7 | 86 ± 9 | 2 ± 11 | 86 ± 6 | 87 ± 6 | 1 ± 7 | 82 ± 6 | 83 ± 9 | 1±10 | 88 ± 8 | 85 ± 10 | -3 ± 12 |
| **Insulin (U/L)** | 5.0 (5.1)^bcd^ | 9.9 (8.8) | 5.5 (7.5)* | 6.8 (7.5)^a^ | 11.1 (10.9) | 3.2 (5.4) | 7.9 (6.9) ^a^ | 12.3 (6.1) | 5.1 (9.7)* | 11.0 (7.2)^a^ | 17.9 (12.0) | 5.3 (14.7) |
| **HOMA-IR** | 1.05 (0.99)^bcd^ | 2.23 (1.67) | 1.49 (1.36)* | 1.41 (1.58)^a^ | 2.47 (2.22) | 0.75 (1.08) | 1.36 (1.56)^a^ | 2.52 (1.45) | 1.03 (2.17) | 2.17 (1.58)^a^ | 4.55 (2.29) | 1.07 (2.88) |
| **Cholesterol (mg/dL)** | 161 (50) | 151 (32) | -13 (26) | 160 (24) | 146 (42) | -26 (27) | 173 (45) | 160 (32) | -9 (30) | 185 (47) | 171 (44) | -20 (15) |
| **LDL-c (mg/dL)** | 90 (36) | 86 (21) | -3 (29) | 95 (33) | 87 (26) | -13 (13) | 105 (30) | 96 (28) | -7 (21) | 107 (40) | 112 (35) | -21 (16) |
| **HDL-c (mg/dL)** | 59 (26)^d^ | 52 (20) | -5 (18) | 60 (20)^d^ | 44 (9) | -12 (13)* | 51 (15) | 45 (8) | -5 (9) | 42 (8)^ab^ | 40 (10) | -4 (8) |
| **TAG (mg/dL)** | 52 (16)^d^ | 66 (28) | 19 (28)* | 59 (27) | 76 (37) | 11 (30) | 55 (31) | 78 (55) | 18 (54)* | 101 (22)^a^ | 110 (55) | 10 (73) |
| **CRP (mg/L)** | 0.30 (0.80)^c^ | 0.70 (1.23) | 0.13 (1.15) | 1.30 (1.65) | 1.58 (3.94) | 0.47 (2.62) | 2.10 (2.40)^a^ | 1.20 (5.16) | 0.50 (4.04) | 0.90 (2.00) | 1.90 (9.53) | 1.00 (5.90) |
| **Leptin (ug/L)** | 1.76 (2.11)^bcd^ | 4.49 (7.01) | 1.40 (4.45)* | 10.02 (4.09)^a^ | 11.73 (6.78) | 1.89 (7.38) | 13.30 (12.44)^a^ | 10.99 (11.49) | 2.47 (16.03) | 21.71 (16.74)^a^ | 17.14 (6.60) | -8.15 (15.44) |
| **TNF-α (ng/L)** | 2.81 (2.12) | 2.41 (0.82) | -0.38 (2.26) | 1.81 (1.93)^c,d^ | 2.46 (1.02) | 0.51 (1.60)† | 3.45 (1.59)^b^ | 2.34 (0.50) | -1.09 (1.39)*† | 3.06 (2.79)^b^ | 2.91 (1.24) | -0.91 (1.86) |
| **IL8 (ng/L)** | 1.46 (1.27) | 1.98 (1.65) | 0.73 (2.07) | 1.23 (1.77) | 2.62 (1.84) | 1.06 (1.95) | 1.58 (0.98) | 2.51 (1.35) | 0.87 (1.82) | 1.80 (0.58) | 2.33 (1.33) | 0.39 (1.02) |
| **Resistin (ug/L)** | 21.99 (20.39) | 16.99 (7.09) | -3.15 (13.08) | 19.23 (15.18) | 20.37 (9.76) | -1.92 (11.64) | 16.27 (15.58) | 21.00 (4.51) | 5.49 (16.78)* | 21.29 (8.68) | 21.88 (8.49) | 3.06 (8.39) |
| **Adiponectin (mg/L)** | 16.59 (14.73) | 11.10 (11.07) | -5.30 (13.69)* | 12.61 (8.80) | 6.28 (5.22) | -6.28 (8.06)* | 13.97 (10.95) | 8.54 (7.21) | -4.60 (9.66)* | 15.05 (10.44) | 9.05 (6.99) | -4.21 (12.80) |
| **MPO (ug/L)** | 17.40 (35.71) | 16.80 (22.52) | -4.88 (40.43) | 39.97 (55.94) | 22.59 (100.08) | 1.63 (164.78) | 17.40 (29.60) | 43.26 (90.52) | 15.23 (106.01) | 30.54 (16.36) | 22.38 (25.76) | -11.93 (56.21) |
| **MCP1 (ng/L)** | 83.92 (39.96) | 112.61 (32.55) | 31.77 (50.25) | 78.92 (47.33) | 97.44 (53.29) | 16.65 (56.84) | 104.19 (48.26) | 114.70 (36.65) | 3.25 (42.71) | 127.75 (44.32) | 107.13 (52.62) | -9.82 (45.99) |
| **tPAI (ug/L)** | 11.35 (12.90)^cd^ | 10.24 (6.06) | -1.52 (14.20) | 14.55 (18.22) | 13.53 (8.27) | -2.79 (13.17) | 19.69 (15.36)^a^ | 21.81 (17.55) | -5.47 (22.32) | 31.73 (10.54)^a^ | 20.20 (12.36) | -12.73 (9.27) |
| **sICAM1 (mg/L)** | 0.11 (0.09) | 0.07 (0.03) | -0.01 (0.08) | 0.09 (0.06) | 0.09 (0.03) | -0.01 (0.03) | 0.11 (0.10) | 0.09 (0.04) | 0.01 (0.14) | 0.11 (0.05) | 0.09 (0.02) | -0.01 (0.06) |

Statistics presented: Mean ± SD; n (%), median (interquartile range). Pre-pub evolution, group differences in prepuberty and in evolution (in text) were controlled for sex and Tanner stage. P-values are adjusted for false discovery rate using Benjamini–Yekutieli procedure.

* Significant change from pre to pubertal stage within group; ^a^significant difference from Normal Weight; ^b^significant difference from overweight; ^c^significant difference from Obesity No-MS; ^d^significant difference from Obesity MS. ☨Increment differences between evolution groups (p<0.005).

*Abbreviations: BMI, body mass index; NW, normal weight, OW, overweight; OB, obesity; WC, waist circumference; SBP, systolic blood pressure; DBP, diastolic blood pressure; HOMA-IR, homeostasis model assessment for insulin resistance; LDL-c, low-density lipoproteins-cholesterol; HDL-c, high-density lipoproteins-cholesterol; TAG, triglycerides; CRP, C-reactive protein; TNF-α: tumoral necrosis factor-alpha; IL8: interleukin 8; MPO: myeloperoxidase; MCP1: Monocyte chemoattractant protein-1; tPAI: total plasminogen activator inhibitor; sICAM: soluble intercellular adhesion molecule-1.*

**Table 3**. Pre and pubertal levels and change of body composition and cardiometabolic risk markers grouped by the presence or absence of metabolic syndrome in puberty.

| Variable | No MS in Puberty (n = 66) | | | MS in Puberty (n = 9) | | |  |
| --- | --- | --- | --- | --- | --- | --- | --- |
|  | Pre | Pub | Increment | Pre | Pub | Increment | p-value |
| **Age (years)** | 7.8 ± 2.1 | 14.6 ± 1.8 | 6.7 ± 2.8* | 7.9 ± 2.4 | 14.4 ± 1.6 | 6.5 ± 2.7* | 0.999 |
| **Tanner stage** | 0.0 ± 0.0 | 4.3 ± 1.0 | 4.3 ± 1.0* | 0.0 ± 0.0 | 4.3 ± 1.0 | 4.3 ± 1.0* | 0.999 |
| **BMI (kg/m2)** | 21.0 (7.1) | 25.2 (7.1) | 3.9 (4.8)* | 26.8 (3.2) | 34.9 (8.0) | 8.0 (4.4)* | 0.009 |
| **BMI-z** | 1.41 (2.69) | 1.28 (2.08) | -0.04 (1.12) | 3.83 (3.45) | 3.20 (2.40) | 0.10 (0.96) | 0.010 |
| **WC (cm)** | 68 (19) | 80 (20) | 14 (15)* | 82 (13) | 110 (10) | 23 (4)* | 0.028 |
| **SBP (mm Hg)** | 102 (14) | 113 (15) | 9 (20) | 110 (17) | 125 (17) | 25 (21) | 0.999 |
| **DBP (mm Hg)** | 62 (10) | 65 (8) | 5 (14) | 64 (13) | 82 (10) | 18 (9)* | 0.999 |
| **Glucose (mg/dL)** | 84 ± 7 | 85 ± 8 | 1 ± 9 | 90 ± 7 | 88 ± 10 | -1 ± 15 | 0.290 |
| **Insulin (U/L)** | 6.4 (5.1) | 11.1 (8.5) | 4.3 (8.2)* | 14.0 (7.1) | 20.9 (11.9) | 9.8 (6.2) | 0.010 |
| **HOMA-IR** | 1.28 (1.17) | 2.28 (1.82) | 0.93 (1.46)* | 3.00 (2.28) | 3.92 (3.23) | 1.48 (2.11) | 0.009 |
| **Cholesterol (mg/dL)** | 167 (43) | 152 (40) | -18 (32)* | 180 (47) | 160 (32) | -20 (22) | 0.999 |
| **LDL-c (mg/dL)** | 101 (32) | 88 (28) | -11 (26) | 120 (37) | 107 (37) | -13 (15) | 0.999 |
| **HDL-c (mg/dL)** | 55 (18) | 48 (13) | -7 (15)* | 43 (7) | 41 (6) | -7 (4) | 0.087 |
| **TAG (mg/dL)** | 52 (21) | 72 (37) | 13 (36)* | 99 (25) | 111 (40) | 24 (29) | 0.153 |
| **Inflammatory biomarkers** | | | | | | | |
| **CRP (mg/L)** | 1.04 (2.47) | 1.20 (3.29) | 0.42 (2.79) | 1.20 (1.13) | 1.15 (5.60) | 0.49 (5.55) | 0.999 |
| **Leptin (ug/L)** | 8.48 (9.63) | 9.25 (9.69) | 1.35 (7.22) | 21.42 (10.49) | 19.69 (15.34) | -7.41 (13.25) | 0.002 |
| **TNF-α (ng/L)** | 2.86 (2.42) | 2.41 (0.88) | -0.62 (2.04) | 2.77 (1.84) | 2.45 (0.97) | -0.73 (1.19) | 0.999 |
| **IL8 (ng/L)** | 1.60 (1.19) | 2.51 (1.65) | 0.89 (2.11)* | 1.35 (0.65) | 2.12 (0.56) | 0.39 (1.55) | 0.999 |
| **Resistin (ug/L)** | 19.23 (16.33) | 19.64 (7.66) | 1.05 (11.52) | 21.29 (17.09) | 21.00 (6.97) | -2.83 (18.86) | 0.999 |
| **Adiponectin (mg/L)** | 14.09 (10.76) | 8.84 (8.82) | -5.62 (12.18)* | 17.75 (14.78) | 8.54 (5.15) | -6.99 (8.59) | 0.999 |
| **MPO (ug/L)** | 23.24 (38.78) | 23.43 (69.28) | 1.94 (81.86) | 28.85 (18.44) | 31.97 (38.70) | 4.25 (55.33) | 0.999 |
| **MCP1 (ng/L)** | 91.75 (56.80) | 109.38 (38.23) | 8.01 (54.31) | 117.09 (23.56) | 114.70 (70.10) | 17.78 (61.32) | 0.999 |
| **tPAI (ug/L)** | 16.30 (14.26) | 13.19 (14.82) | -3.07 (19.49) | 37.80 (23.83) | 20.20 (17.09) | -10.98 (16.58) | 0.010 |
| **sICAM1 (mg/L)** | 0.11 (0.10) | 0.08 (0.03) | -0.01 (0.08) | 0.10 (0.05) | 0.09 (0.24) | 0.02 (0.21) | 0.031 |

Statistics presented: Mean ± SD; n (%), median (interquartile range). Pre-pub evolution, group differences in prepuberty and in evolution (in text) were controlled for sex and Tanner stage. P-values are adjusted for false discovery rate using Benjamini–Yekutieli procedure.

* Significant change from pre to pubertal stage within group.

*Abbreviations: BMI, body mass index; NW, normal weight, OW, overweight; OB, obesity; WC, waist circumference; SBP, systolic blood pressure; DBP, diastolic blood pressure; HOMA-IR, homeostasis model assessment for insulin resistance; LDL-c, low-density lipoproteins-cholesterol; HDL-c, high-density lipoproteins-cholesterol; TAG, triglycerides; CRP, C-reactive protein; TNF-α: tumoral necrosis factor-alpha; IL8: interleukin 8; MPO: myeloperoxidase; MCP1: Monocyte chemoattractant protein-1; tPAI: total plasminogen activator inhibitor; sICAM: soluble intercellular adhesion molecule-1.*

**Table 4**. Logistic model for predict prevalence of Metabolic Syndrome in puberty for participants with obesity in prepuberty.

|  | *Metabolic syndrome in puberty* | | | | |
| --- | --- | --- | --- | --- | --- |
| *Predictors* | *OR* | *OR 95% CI* | *β* | *β 95% CI* | *p* |
| (Intercept) | 0.00 | 0.00 – 0.00 | 0.14 | 0.02 – 0.49 | 0.020 |
| Age prepubertal | 4.37 | 1.34 – 24.44 | 20.70 | 1.82 – 711.28 | 0.037 |
| Tanner Prepubertal | 2.71 | 0.72 – 14.66 | 2.82 | 0.71 – 16.24 | 0.176 |
| BMI-Z Prepuberal | 4.27 | 1.39 – 22.59 | 13.56 | 1.81 – 271.04 | 0.034 |
| Leptin Prepubertal | 0.83 | 0.64 – 0.98 | 0.11 | 0.01 – 0.84 | 0.062 |
| tPAI Prepubertal | 1.19 | 1.06 – 1.43 | 10.58 | 2.10 – 118.15 | 0.017 |
| HOMA-IR Prepubertal | 2.28 | 1.02 – 6.35 | 3.95 | 1.03 – 21.89 | 0.059 |

CI = Confidence interval; BMI-Z = BMI Z-score; tPAI = tissue plasminogen activator inhibitor; HOMA-IR = Homeostatic Model Assessment of Insulin Resistance.

Backwards stepwise model selection based on AIC was used to select the final model that best predict MS in puberty for participants with obesity in prepuberty. Variables included in the initial model were prepubertal levels of BMI-z, Leptin, TNF-α, Resistin, Adiponectin, sICAM1, MPO, MCP1, tPAI, HOMA-IR and age, as well as sex and Tanner stage in puberty.

**Supplementary table 1.** F-test and pairwise comparisons for prepubertal-pubertal change, controlled for tanner stage and sex

| F-tests | | | | Within-group change | | | | Group | Between-group prepuber | | | Between-group diff change | | |
| --- | --- | --- | --- | --- | --- | --- | --- | --- | --- | --- | --- | --- | --- | --- |
| Term | F | p | p (adj) | NW | OW | OB | OBms |  | NW | OW | OB | NW | OW | OB |
| **Adiponectin** | | | | | | | | | | | | | | |
| Group | 0.60 | 0.616 | >0.999 | **0.047** | **0.005** | **0.047** | 0.625 | OW | >0.999 |  |  | >0.999 |  |  |
| Stage | 39.18 | **<0.001** | **<0.001** |  |  |  |  | OB | >0.999 | >0.999 |  | >0.999 | >0.999 |  |
| Group × Stage | 0.75 | 0.524 | >0.999 |  |  |  |  | OBms | >0.999 | >0.999 | >0.999 | >0.999 | >0.999 | >0.999 |
| **Age** | | | | | | | | | | | | | | |
| Group | 2.36 | 0.074 | 0.732 | **<0.001** | **<0.001** | **<0.001** | **<0.001** | OW | 0.609 |  |  | >0.999 |  |  |
| Stage | 528.37 | **<0.001** | **<0.001** |  |  |  |  | OB | 0.395 | **0.022** |  | >0.999 | 0.595 |  |
| Group × Stage | 1.52 | 0.213 | >0.999 |  |  |  |  | OBms | >0.999 | >0.999 | 0.500 | >0.999 | >0.999 | >0.999 |
| **BMI-Z** | | | | | | | | | | | | | | |
| Group | 94.61 | **<0.001** | **<0.001** | 0.590 | >0.999 | **0.004** | >0.999 | OW | **<0.001** |  |  | >0.999 |  |  |
| Stage | 0.00 | 0.997 | >0.999 |  |  |  |  | OB | **<0.001** | **<0.001** |  | **0.005** | **0.013** |  |
| Group × Stage | 5.85 | **0.001** | **0.019** |  |  |  |  | OBms | **<0.001** | **<0.001** | >0.999 | >0.999 | >0.999 | 0.205 |
| **Cholesterol** | | | | | | | | | | | | | | |
| Group | 0.44 | 0.726 | >0.999 | 0.396 | 0.110 | 0.527 | 0.396 | OW | >0.999 |  |  | >0.999 |  |  |
| Stage | 16.32 | **<0.001** | **0.002** |  |  |  |  | OB | >0.999 | >0.999 |  | >0.999 | >0.999 |  |
| Group × Stage | 0.63 | 0.596 | >0.999 |  |  |  |  | OBms | >0.999 | >0.999 | >0.999 | >0.999 | >0.999 | >0.999 |
| **CRP** | | | | | | | | | | | | | | |
| Group | 4.17 | **0.009** | 0.105 | 0.519 | >0.999 | >0.999 | 0.519 | OW | 0.068 |  |  | >0.999 |  |  |
| Stage | 1.92 | 0.170 | >0.999 |  |  |  |  | OB | **0.015** | >0.999 |  | >0.999 | >0.999 |  |
| Group × Stage | 1.00 | 0.399 | >0.999 |  |  |  |  | OBms | 0.907 | >0.999 | 0.907 | >0.999 | >0.999 | >0.999 |
| **DBP** | | | | | | | | | | | | | | |
| Group | 11.64 | **<0.001** | **<0.001** | >0.999 | **0.027** | **0.027** | >0.999 | OW | 0.286 |  |  | 0.330 |  |  |
| Stage | 20.97 | **<0.001** | **<0.001** |  |  |  |  | OB | >0.999 | 0.555 |  | 0.330 | >0.999 |  |
| Group × Stage | 2.38 | 0.072 | 0.732 |  |  |  |  | OBms | **<0.001** | **<0.001** | **<0.001** | >0.999 | 0.330 | 0.330 |
| **Glucose** | | | | | | | | | | | | | | |
| Group | 1.48 | 0.228 | >0.999 | 0.838 | 0.838 | 0.838 | >0.999 | OW | >0.999 |  |  | >0.999 |  |  |
| Stage | 1.65 | 0.203 | >0.999 |  |  |  |  | OB | >0.999 | >0.999 |  | >0.999 | >0.999 |  |
| Group × Stage | 0.25 | 0.858 | >0.999 |  |  |  |  | OBms | >0.999 | >0.999 | >0.999 | >0.999 | >0.999 | >0.999 |
| **HDL** | | | | | | | | | | | | | | |
| Group | 7.83 | **<0.001** | **0.003** | 0.220 | **0.010** | 0.311 | 0.754 | OW | >0.999 |  |  | >0.999 |  |  |
| Stage | 17.93 | **<0.001** | **0.001** |  |  |  |  | OB | 0.058 | 0.264 |  | >0.999 | >0.999 |  |
| Group × Stage | 1.04 | 0.379 | >0.999 |  |  |  |  | OBms | **<0.001** | **0.005** | 0.094 | >0.999 | >0.999 | >0.999 |
| **HOMA-IR** | | | | | | | | | | | | | | |
| Group | 9.10 | **<0.001** | **<0.001** | **<0.001** | 0.061 | **0.001** | 0.092 | OW | **0.023** |  |  | >0.999 |  |  |
| Stage | 20.35 | **<0.001** | **<0.001** |  |  |  |  | OB | **0.022** | >0.999 |  | >0.999 | >0.999 |  |
| Group × Stage | 0.38 | 0.767 | >0.999 |  |  |  |  | OBms | **0.001** | 0.299 | 0.283 | >0.999 | >0.999 | >0.999 |
| **IL8** | | | | | | | | | | | | | | |
| Group | 0.19 | 0.902 | >0.999 | 0.242 | 0.210 | 0.521 | >0.999 | OW | >0.999 |  |  | >0.999 |  |  |
| Stage | 8.60 | **0.004** | 0.055 |  |  |  |  | OB | >0.999 | >0.999 |  | >0.999 | >0.999 |  |
| Group × Stage | 0.66 | 0.582 | >0.999 |  |  |  |  | OBms | >0.999 | >0.999 | >0.999 | >0.999 | >0.999 | >0.999 |
| **Insulin** | | | | | | | | | | | | | | |
| Group | 10.16 | **<0.001** | **<0.001** | **0.001** | 0.076 | **0.001** | 0.078 | OW | **0.022** |  |  | >0.999 |  |  |
| Stage | 23.56 | **<0.001** | **<0.001** |  |  |  |  | OB | **0.010** | >0.999 |  | >0.999 | >0.999 |  |
| Group × Stage | 0.37 | 0.772 | >0.999 |  |  |  |  | OBms | **0.001** | 0.310 | 0.310 | >0.999 | >0.999 | >0.999 |
| **LDL** | | | | | | | | | | | | | | |
| Group | 1.83 | 0.149 | >0.999 | 0.545 | 0.448 | 0.545 | 0.545 | OW | >0.999 |  |  | >0.999 |  |  |
| Stage | 13.45 | **<0.001** | **0.008** |  |  |  |  | OB | 0.850 | >0.999 |  | >0.999 | >0.999 |  |
| Group × Stage | 0.23 | 0.873 | >0.999 |  |  |  |  | OBms | 0.850 | 0.850 | >0.999 | >0.999 | >0.999 | >0.999 |
| **Leptin** | | | | | | | | | | | | | | |
| Group | 42.15 | **<0.001** | **<0.001** | **0.039** | >0.999 | 0.928 | 0.772 | OW | **<0.001** |  |  | 0.533 |  |  |
| Stage | 0.61 | 0.436 | >0.999 |  |  |  |  | OB | **<0.001** | 0.240 |  | **0.048** | 0.821 |  |
| Group × Stage | 3.92 | **0.012** | 0.136 |  |  |  |  | OBms | **<0.001** | 0.065 | 0.647 | **0.048** | 0.533 | >0.999 |
| **MCP1** | | | | | | | | | | | | | | |
| Group | 2.34 | 0.081 | 0.773 | >0.999 | >0.999 | >0.999 | >0.999 | OW | 0.832 |  |  | >0.999 |  |  |
| Stage | 4.48 | **0.037** | 0.406 |  |  |  |  | OB | 0.929 | 0.268 |  | >0.999 | >0.999 |  |
| Group × Stage | 0.51 | 0.680 | >0.999 |  |  |  |  | OBms | 0.929 | 0.268 | >0.999 | >0.999 | >0.999 | >0.999 |
| **MPO** | | | | | | | | | | | | | | |
| Group | 0.81 | 0.489 | >0.999 | >0.999 | >0.999 | 0.746 | 0.746 | OW | >0.999 |  |  | >0.999 |  |  |
| Stage | 0.02 | 0.899 | >0.999 |  |  |  |  | OB | >0.999 | >0.999 |  | 0.849 | 0.849 |  |
| Group × Stage | 1.52 | 0.212 | >0.999 |  |  |  |  | OBms | >0.999 | >0.999 | >0.999 | 0.849 | 0.849 | 0.621 |
| **Resistin** | | | | | | | | | | | | | | |
| Group | 0.58 | 0.630 | >0.999 | >0.999 | >0.999 | >0.999 | >0.999 | OW | >0.999 |  |  | >0.999 |  |  |
| Stage | 2.06 | 0.155 | >0.999 |  |  |  |  | OB | 0.642 | >0.999 |  | >0.999 | >0.999 |  |
| Group × Stage | 1.15 | 0.335 | >0.999 |  |  |  |  | OBms | >0.999 | >0.999 | >0.999 | >0.999 | >0.999 | >0.999 |
| **SBP** | | | | | | | | | | | | | | |
| Group | 5.13 | **0.003** | **0.039** | >0.999 | 0.511 | **<0.001** | >0.999 | OW | >0.999 |  |  | >0.999 |  |  |
| Stage | 23.42 | **<0.001** | **<0.001** |  |  |  |  | OB | >0.999 | >0.999 |  | **0.025** | 0.161 |  |
| Group × Stage | 4.58 | **0.006** | 0.067 |  |  |  |  | OBms | **0.015** | 0.072 | **0.015** | >0.999 | >0.999 | 0.058 |
| **sICAM1** | | | | | | | | | | | | | | |
| Group | 0.52 | 0.669 | >0.999 | >0.999 | 0.881 | 0.611 | 0.881 | OW | >0.999 |  |  | >0.999 |  |  |
| Stage | 0.43 | 0.514 | >0.999 |  |  |  |  | OB | >0.999 | >0.999 |  | >0.999 | >0.999 |  |
| Group × Stage | 0.41 | 0.748 | >0.999 |  |  |  |  | OBms | >0.999 | >0.999 | >0.999 | >0.999 | >0.999 | >0.999 |
| **TAG** | | | | | | | | | | | | | | |
| Group | 5.39 | **0.002** | **0.030** | **0.007** | 0.069 | **0.005** | 0.973 | OW | 0.211 |  |  | >0.999 |  |  |
| Stage | 13.51 | **<0.001** | **0.008** |  |  |  |  | OB | 0.280 | >0.999 |  | >0.999 | >0.999 |  |
| Group × Stage | 0.63 | 0.600 | >0.999 |  |  |  |  | OBms | **0.002** | 0.096 | 0.051 | >0.999 | >0.999 | >0.999 |
| **TNF** | | | | | | | | | | | | | | |
| Group | 2.41 | 0.074 | 0.732 | >0.999 | 0.333 | **<0.001** | 0.397 | OW | 0.136 |  |  | 0.372 |  |  |
| Stage | 11.05 | **0.001** | **0.019** |  |  |  |  | OB | 0.112 | **<0.001** |  | **0.049** | **<0.001** |  |
| Group × Stage | 6.63 | **<0.001** | **0.008** |  |  |  |  | OBms | 0.265 | **0.011** | >0.999 | 0.774 | 0.104 | 0.857 |
| **tPAI** | | | | | | | | | | | | | | |
| Group | 8.65 | **<0.001** | **0.001** | 0.425 | >0.999 | >0.999 | 0.425 | OW | 0.322 |  |  | >0.999 |  |  |
| Stage | 2.17 | 0.144 | >0.999 |  |  |  |  | OB | **0.047** | 0.832 |  | >0.999 | >0.999 |  |
| Group × Stage | 0.64 | 0.593 | >0.999 |  |  |  |  | OBms | **0.003** | 0.081 | 0.287 | >0.999 | >0.999 | >0.999 |
| **WC** | | | | | | | | | | | | | | |
| Group | 55.25 | **<0.001** | **<0.001** | **<0.001** | **<0.001** | **<0.001** | **<0.001** | OW | **<0.001** |  |  | >0.999 |  |  |
| Stage | 130.85 | **<0.001** | **<0.001** |  |  |  |  | OB | **<0.001** | **0.002** |  | >0.999 | >0.999 |  |
| Group × Stage | 0.08 | 0.968 | >0.999 |  |  |  |  | OBms | **<0.001** | **<0.001** | 0.383 | >0.999 | >0.999 | >0.999 |
| Group = Obesity status at prepubertal stage; Stage = Pubertal stage (prepubetal, pubertal); NW = Normal Weight; OW = Overweight; OB = Obese no metabolic syndrome; OBms = Obese with metabolic syndrome; BMI-Z = BMI Z-score; Sum SF = Sum of skinfolds; WC = Waist circumference, WHR = Waist-hip ratio; SBP = Systolic blood pressure; DBP = Diastolic blood pressure; LDL = Low-density lipoprotein; HDL = High-density lipoprotein; TAG = triacylglycerol; HOMA-IR = Homeostatic Model Assessment of Insulin Resistance. | | | | | | | | | | | | | | |

**Supplementary table 2**. Stepwise logistic regression to predict prevalence of metabolic syndrome in pubertal stage within participants with obesity in prepubertal stage.

|  | Step 1 | Step 2 | Step 3 | Step 4 | Step 5 | Step 6 | Step 7 | Step 8 | Step 9 | Step 10 | Step 11 | Step 12 |
| --- | --- | --- | --- | --- | --- | --- | --- | --- | --- | --- | --- | --- |
| (Intercept) | -23.97 | -24.45 | -24.14 | -22.60 | -21.23 | -18.55 | -18.35 | -16.33 | -12.96 | -10.83 | -7.47 | -7.34 |
| CI | [-62.21, -0.94] | [-59.75, -2.95] | [-58.67, -2.92] | [-51.68, -2.98] | [-50.44, -2.81] | [-44.05, -3.81] | [-43.27, -3.90] | [-35.93, -4.67] | [-25.46, -4.37] | [-19.71, -4.40] | [-12.20, -4.47] | [-12.02, -4.35] |
| BMI-Z pre | 1.27 | 1.26 | 1.25 | 1.24 | 1.07 | 1.09 | 1.07 | 1.15 | 0.97 | 0.76 | 0.58 | 0.50 |
| CI | [-0.07, 3.21] | [-0.07, 3.18] | [-0.07, 3.12] | [-0.05, 3.06] | [0.00, 2.61] | [0.02, 2.64] | [0.02, 2.57] | [0.23, 2.61] | [0.17, 2.02] | [0.23, 1.48] | [0.15, 1.12] | [0.09, 1.02] |
| Leptin pre | -0.10 | -0.11 | -0.11 | -0.10 | -0.09 | -0.07 | -0.07 | -0.06 | -0.05 |  |  |  |
| CI | [-0.40, 0.11] | [-0.39, 0.07] | [-0.38, 0.07] | [-0.31, 0.07] | [-0.30, 0.08] | [-0.27, 0.08] | [-0.26, 0.08] | [-0.22, 0.08] | [-0.20, 0.08] |  |  |  |
| TNF-α pre | -0.04 |  |  |  |  |  |  |  |  |  |  |  |
| CI | [-1.36, 1.18] |  |  |  |  |  |  |  |  |  |  |  |
| Resistin pre | 0.02 | 0.02 | 0.02 |  |  |  |  |  |  |  |  |  |
| CI | [-0.13, 0.15] | [-0.12, 0.15] | [-0.11, 0.15] |  |  |  |  |  |  |  |  |  |
| Adiponectin pre | 0.06 | 0.06 | 0.07 | 0.07 | 0.07 | 0.07 | 0.06 | 0.05 |  |  |  |  |
| CI | [-0.12, 0.26] | [-0.12, 0.26] | [-0.08, 0.23] | [-0.08, 0.23] | [-0.08, 0.23] | [-0.08, 0.23] | [-0.08, 0.22] | [-0.09, 0.19] |  |  |  |  |
| sICAM1 pre | 6.60 | 6.75 | 6.77 | 5.68 | 5.69 |  |  |  |  |  |  |  |
| CI | [-25.05, 27.58] | [-24.92, 27.17] | [-24.23, 27.04] | [-24.32, 23.18] | [-24.39, 23.41] |  |  |  |  |  |  |  |
| MPO pre | 0.01 | 0.01 | 0.01 | 0.01 | 0.01 | 0.01 | 0.01 | 0.01 | 0.01 | 0.01 | 0.01 |  |
| CI | [-0.02, 0.05] | [-0.02, 0.05] | [-0.02, 0.05] | [0.00, 0.04] | [0.00, 0.04] | [0.00, 0.03] | [0.00, 0.03] | [0.00, 0.03] | [0.00, 0.03] | [0.00, 0.03] | [0.00, 0.03] |  |
| MCP1 pre | -0.01 | -0.01 | -0.01 | -0.01 | -0.01 | -0.01 |  |  |  |  |  |  |
| CI | [-0.04, 0.02] | [-0.04, 0.02] | [-0.04, 0.02] | [-0.04, 0.02] | [-0.04, 0.02] | [-0.03, 0.02] |  |  |  |  |  |  |
| tPAI pre | 0.12 | 0.12 | 0.12 | 0.11 | 0.11 | 0.11 | 0.10 | 0.10 | 0.09 | 0.08 | 0.07 | 0.08 |
| CI | [0.00, 0.33] | [0.00, 0.32] | [0.01, 0.31] | [0.01, 0.26] | [0.01, 0.26] | [0.01, 0.26] | [0.01, 0.25] | [0.00, 0.23] | [0.01, 0.21] | [0.00, 0.17] | [-0.01, 0.15] | [0.01, 0.16] |
| HOMA-IR pre | 0.49 | 0.50 | 0.51 | 0.49 | 0.51 | 0.48 | 0.46 | 0.42 | 0.47 | 0.44 | 0.59 | 0.64 |
| CI | [-0.29, 1.41] | [-0.19, 1.37] | [-0.15, 1.36] | [-0.16, 1.25] | [-0.13, 1.28] | [-0.15, 1.23] | [-0.17, 1.19] | [-0.18, 1.09] | [-0.11, 1.13] | [-0.13, 1.04] | [0.11, 1.14] | [0.15, 1.19] |
| Age pre | 0.97 | 0.97 | 0.97 | 0.93 | 0.76 | 0.73 | 0.73 | 0.83 | 0.57 | 0.36 |  |  |
| CI | [-0.50, 2.81] | [-0.47, 2.81] | [-0.47, 2.80] | [-0.48, 2.69] | [-0.43, 2.29] | [-0.47, 2.28] | [-0.46, 2.28] | [-0.22, 2.32] | [-0.28, 1.61] | [-0.26, 1.04] |  |  |
| Tanner pub | 0.10 | 0.10 |  |  |  |  |  |  |  |  |  |  |
| CI | [-1.54, 1.84] | [-1.54, 1.82] |  |  |  |  |  |  |  |  |  |  |
| Sex × Female | 0.64 | 0.62 | 0.65 | 0.70 |  |  |  |  |  |  |  |  |
| CI | [-2.69, 4.34] | [-2.67, 4.27] | [-2.62, 4.26] | [-2.51, 4.28] |  |  |  |  |  |  |  |  |
| WC pre | 0.06 | 0.07 | 0.07 | 0.06 | 0.07 | 0.04 | 0.04 |  |  |  |  |  |
| CI | [-0.17, 0.32] | [-0.15, 0.31] | [-0.14, 0.31] | [-0.14, 0.28] | [-0.13, 0.29] | [-0.14, 0.22] | [-0.14, 0.21] |  |  |  |  |  |
| Num.Obs. | 75 | 75 | 75 | 75 | 75 | 75 | 75 | 75 | 75 | 75 | 75 | 75 |
| AIC | 55.5 | 53.5 | 51.5 | 49.6 | 47.8 | 46.0 | 44.3 | 42.4 | 41.0 | 39.5 | 38.8 | 38.7 |
| BIC | 90.2 | 85.9 | 81.6 | 77.4 | 73.3 | 69.2 | 65.1 | 61.0 | 57.2 | 53.4 | 50.4 | 48.0 |
| Log likehood | -12.735 | -12.738 | -12.747 | -12.791 | -12.888 | -13.011 | -13.131 | -13.223 | -13.509 | -13.744 | -14.390 | -15.357 |

CI: Conficence interval. AIC: Akaike Information Criteria. BIC: Bayes Information Criterion,
